# Lovastatin reduces PEL cell survival by phosphorylating ERK1/2 that blocks the autophagic flux and engages a cross‐talk with p53 to activate p21

**DOI:** 10.1002/iub.2503

**Published:** 2021-05-21

**Authors:** Roberta Santarelli, Chiara Pompili, Maria Saveria Gilardini Montani, Maria Anele Romeo, Roberta Gonnella, Gabriella D'Orazi, Mara Cirone

**Affiliations:** ^1^ Department of Experimental Medicine “Sapienza” University of Rome, Laboratory affiliated to Istituto Pasteur Italia‐Fondazione Cenci Bolognetti Rome Italy; ^2^ Translational Research Area Regina Elena National Cancer Institute Rome Italy; ^3^ Department of Medical Oral and Biotechnological Sciences, University “G. d'Annunzio” Chieti Italy

**Keywords:** apoptosis, autophagy, ERK1/2, lovastatin, p21, p53, PEL, STAT3, statins

## Abstract

Statins are inhibitors of the mevalonate pathway that besides being cholesterol lowering agents, display anti‐cancer properties. This is because cholesterol is an essential component of cell membranes but also because the mevalonate pathway controls protein farnesylation and geranylation, processes essential for the activity of GTPase family proteins. In this study, we found that Lovastatin exerted a dose‐ and time‐dependent cytotoxic effect against PEL cells, an aggressive B cell lymphoma strictly associated with the gammaherpesvirus KSHV and characterized by a poor response to conventional chemotherapies. At molecular level, Lovastatin by dephosphorylating STAT3, induced ERK1/2 activation that inhibited autophagy and phosphorylated p53ser15 that in turn maintained ERK1/2 activated and up‐regulated p21. However, p21 played a pro‐survival role in this setting, as its inhibition by UC2288 further reduced cell survival in PEL cells undergoing Lovastatin treatment. In conclusion, this study suggests that Lovastatin may represent a valid therapeutic alternative against PEL cells, especially if used in combination with p21 inhibitors.

**Abbreviations**
AMPK: AMP‐activated protein kinase
ATG5: autophagy protein 5
ERK 1/2: extracellular signal‐regulated protein kinase 1/2
HMG‐CoA reductase: 3‐hydroxy‐3‐methyl‐glutaryl‐CoA reductase
KSHV: Kaposi's Sarcoma‐associated Herpesvirus
LC3: microtubule‐associated protein 1A/1B‐light chain 3
mTOR: mammalian target of rapamycin
p21: cyclin‐dependent kinase inhibitor 1
p62: sequestosome 1 (SQSTM1)
PARP1: poly (ADP‐ribose) polymerase 1
PEL: primary effusion lymphoma
STAT3: signal transducer and activator of transcription 3

## 1 INTRODUCTION

Lovastatin that belongs to the class of Statins, is a lactone, a closed ring prodrug, which is transformed into active open forms in the body to act as a 3‐hydroxy‐3‐methyl‐glutaryl‐CoA (HMG‐CoA) reductase inhibitor, interfering with the mevalonate pathway. Statins, besides being cholesterol reducing agents, may affect the GTPase family protein activity^1^ or affect the activation of multiple pathways that control cell survival such as Signal Transducer and activator of Transcription 3 (STAT3), AMP‐activated protein kinase (AMPK)^2^ and Extracellular signal‐regulated protein kinase (ERK) 1/2.^3^ In the case of ERK1/2, either the activation or the inhibition^4^ has been observed to be induced by statin treatment. Statins have been shown to have an anti‐cancer potential against a variety of cancers, including those carrying mutp53.^5^ Indeed, in these cancer types, Statins interrupt the interplay between mutp53 and the mevalonate pathway that strongly sustains cancer cell survival.^6^, ^7^, ^8^ However, Statins can be cytotoxic also against cancers carrying wtp53, in which the activation of p53 contributes to its cytotoxic effect.^9^ Interestingly, the activation of wtp53 may inhibit the mevalonate pathway, differently from what reported for mutp53.^10^

In this study, we explored the anti‐cancer potential of Lovastatin against Primary Effusion Lymphoma (PEL), a B cell lymphoma strictly linked to Kaposi's Sarcoma‐associated Herpesvirus (KSHV) and characterized by a poor response to conventional chemotherapies. PEL cells carry wtp53 and display the constitutive activation of several pro‐survival pathways such as STAT3 and mammalian target of rapamycin (mTOR), whose targeting has been reported to be an effective strategy in reducing PEL cells survival.^11^, ^12^, ^13^ Moreover, as we have previously shown, drugs such as capsaicin,^14^ metformin^15^ or quercetin,^16^ able to concomitantly target multiple pathways activated in PEL, are promising agents in reducing PEL cell survival. Particularly because it has been shown that the activation of these pathways is often inter‐connected.^16^ We also observed that treatments inhibiting some of pro‐survival pathways (i.e., STAT3) may lead to the activation of p53 apoptotic program in PEL cells,^17^, ^18^ confirming previous studies indicating that STAT3 and p53, that have opposite function in cancer survival, can inhibit each other.^19^, ^20^ However, it must be considered that p53 activation may play a role in up‐regulating cyclin‐dependent kinase inhibitor 1 (p21) that, although blocks cell cycle, may allow cancer cells to repair DNA damage and thus prevent cell death.^21^ p21 up‐regulation mainly occurs following p53‐Ser15 phosphorylation, that may be mediated by ERK1/2, among other kinases.^22^ In addition, ERK1/2 may up‐regulate p21 independently of p53.^23^, ^24^

ERK1/2 has been reported to either promote or reduce cancer cell survival,^25^ depending on the cell context in which it is activated. Furthermore, ERK1/2 may have an impact on the autophagic process, although, also in this case, its role seems to be controversial.^26^, ^27^ Based on this knowledge, in this study we investigated the effect of Lovastatin on cell survival and autophagy in PEL cells and explored the molecular pathways activated/inhibited by such treatment.

## 2 EXPERIMENTAL PROCEDURES

### 2.1 Cell culture, reagents and treatments

BC3 (ATCC, CRL‐2277) and BCBL1 (kindly provided by Prof. P. Monini, National AIDS Center, Istituto Superiore di Sanità, Rome, Italy) are human B‐cell lines derived from Primary Effusion Lymphoma (PEL) carrying latent KSHV. Both cell lines were cultured in RPMI‐1640 medium, containing L‐glutamine (2 mM), streptomycin (100 μg/ml) (Corning, NY; 30‐002), penicillin (100 U/ml) (Corning, NY; 25‐005) and supplemented with 10% Fetal Bovine Serum (FBS) (Corning, NY; 35‐079). Cells were grown at 37°C in a 5% CO_2_ incubator.

To test Lovastatin cytotoxicity, BC3 and BCBL1 cells (3 × 10^5^/ml) were cultured in the presence of 3, 10, or 30 μM Lovastatin for 48 h. Untreated cells were used as control (CT) and DMSO as vehicle. Since 30 μM Lovastatin showed the highest cytotoxicity, this concentration was used to perform the next experiments.

Depending on the experiments, BC3 and BCBL1 cell lines (3 × 10^5^/ml) were pre‐treated with PD98059 (20 μM), or UC2288 (5 μM) or with Pifithrin‐α (20 μM) for 45 min before culturing with Lovastatin (30 μM) for additional 24 and 48 h. PD98059 is a cell permeable inhibitor of MAP kinase kinase (MEK) and, consequently, it was used to inhibit ERK1/2 phosphorylation/activation. UC2288 is a p21 inhibitor while Pifithrin‐α inhibits p53. Lovastatin belongs to the Statins, HMG‐CoA reductase inhibitors.

A Trypan blue exclusion assay was carried out to test the cell viability after treatments: live cells were counted by light microscopy using a Neubauer hemocytometer.

To investigate autophagy by fluorescence live‐cell imaging, 3 × 10^5^/ml BC3 cells stably transfected with pEGFP‐LC3 plasmid (GFP‐LC3 BC3) were grown in RPMI supplemented with Geneticin (G418; 0.4 mg/ml) in presence of Lovastatin (30 μM) or vehicle (CT) for 48 h. Cells were pre‐treated or not with PD98059 (20 μM) for 45 min and Cloroquine (CQ) (10 μM) was added for the last 24 h. Cells were then harvested and washed in cold PBS. Pellets were resuspended in PBS: glycerol (1:1) and LC3 dots visualized by fluorescence microscopy (Olympus BX53, USA) at ×20 magnification.

### 2.2 Western blot analysis

Cells were washed twice with phosphate‐buffered saline (PBS; Gibco, 18912‐014) and lysed in a RIPA buffer containing 150 mM NaCl, 1% NP‐40 (Calbiochem, 492015), 50 mM Tris–HCl, pH 8, 0.5% deoxycholic acid (SIGMA, D‐6750), 0.1% SDS (SERVA, 39575.02), 1% Triton X‐100, protease and phosphatase inhibitors (SIGMA, S8830, S6508, and 450022). 10 μg of each lysate were subjected to electrophoresis on 4–12% NuPage Bis‐Tris gels (Life Technologies/Novex, NP0323) and transferred to nitrocellulose membranes (Protran, GE Healthcare/Amersham). The membranes were then blocked in PBS‐0.1% Tween‐20 (SIGMA, P1379) containing 3% BSA (SIGMA, A4503) and probed with specific primary antibodies. After several washes in PBS‐0.1% Tween 20 the membranes were incubated with appropriate secondary antibodies conjugated to horseradish peroxidase. Finally, the membranes were washed in PBS‐0.1% Tween‐20 and immunoreactivity was detected using an enhanced chemiluminescence kit (Thermo Scientific, 32209). All the primary and secondary antibodies were diluted in PBS‐0.1% Tween20 solution containing 3% of BSA (SERVA, Reno, NV; 11,943.03). Densitometric analysis was performed using ImageJ software (http://imagej.nih.gov).

### 2.3 Antibodies

The following primary antibodies were used in western blotting analysis: rabbit polyclonal anti‐PARP1 (1:1000; Proteintech, #13371‐1‐AP); mouse monoclonal anti‐phospho ERK1/2 (1:500; Santa Cruz Biotech, #7383); rabbit polyclonal anti‐ERK1 and rabbit polyclonal anti‐ERK2 (1:500; Santa Cruz Biotech, #sc‐93 and sc‐154, respectively); rabbit polyclonal anti‐phosphoSTAT3 Tyr705 (1:500; Santa Cruz Biotech, #sc‐8059); mouse monoclonal anti‐STAT3 (1:100; BD Transduction Lab, #610189); rabbit polyclonal anti‐p21 (1:200; Santa Cruz Biotech, #sc‐397); rabbit polyclonal anti LC3 I/II (1:1000; Novus, #NB100‐2220); mouse monoclonal anti‐p62/SQSTM1 (1:300, BD Transduction Lab, # 610832); mouse monoclonal anti‐phosphop53Ser15 (1:100; Cell Signaling, #16G8). Mouse monoclonal anti‐β‐Actin (1:10.000 Sigma Aldrich, St Louis, MO; #A5441) was used as loading control. The goat polyclonal anti‐mouse IgG‐Horseradish Peroxidase (HRP) (Santa Cruz Biotechnology, #sc‐2005) and anti‐rabbit IgG‐HRP (Santa Cruz Biotechnology #sc‐2004) were used as secondary antibodies.

### 2.4 Apoptosis and cell cycle analysis

BC3 and BCBL1 cells (3 × 10^5^/ml) were cultured with Lovastatin (30 μM), or with vehicle (CT), for 24 and 48 h. In some experiments, both cell lines were pre‐treated for 45 min with p21 inhibitor UC2288. Cells were then washed and: (1) after lysis PARP1 cleavage (PARPcl) was assessed by western blotting; (2) sub‐G1 cell cycle analysis was performed. At this aim, the DNA content was analyzed using the method of Propidium Iodide (Sigma Aldrich, St Louis, MO; P4170) staining and flow cytometry. Cells were washed with cold ×1 PBS and fixed in 70% ethanol on ice for at least 1 h. Cell pellet was washed three times with cold ×1 PBS and stained with 50 μg/ml PI and RNase for 15 min at 37°C. DNA content was measured by a BD Biosciences FACSCalibur. Cell debris was excluded from analysis by increasing the forward scatter threshold. Cells with a DNA content lower and a Side Scatter higher than that of G0/G1 cells, were considered as apoptotic cells, sub‐G1. Data are representative of at least three independent experiments.

### 2.5 ATG5 silencing

BC3 cells were plated at a density of 8 × 10^5^ cells/well in six well plates, in 1.25 ml of complete medium without antibiotics. ATG5 silencing was performed using Lipofectamine 2000 by transfecting 30 nmol of specific or scrambled (sc) siRNAs (Santa Cruz Biotechnology, sc‐41445 and sc‐37007, respectively), according to the manufacturer's instructions. Then cells were cultured for further 48 h, lysed and protein extracts were analysed by western blotting.

### 2.6 Statistical analysis

Results are represented by the mean ± SD of at least three independent experiments. The Student *t* test was used for statistical significance of the differences between treatment groups. Statistical analysis was performed using analysis of variance at 5% (*p* < .05).

## 3 RESULTS

### 3.1 Lovastatin induces a timedependent cell death in BC3 and BCBL1 PEL cells

**FIGURE 1:**
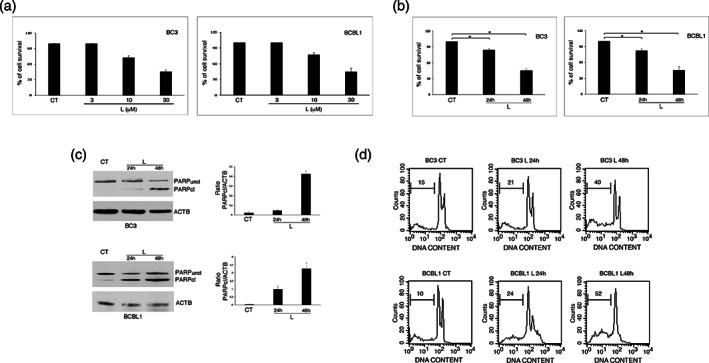
Lovastatin induces apoptosis in PEL cell lines. (a) BC3 and BCBL1 cells were treated or not (CT) with Lovastatin (L) (3‐10‐30 μM) for 48 h and cellular viability was assessed by Trypan blue exclusion. % of cell survival is shown. (b) BC3 and BCBL1 cells were treated or not (CT) with Lovastatin (L) (30 μM) for 24 and 48 h and cellular viability was assessed by Trypan blue exclusion. % of cell survival is shown. * < 0.05. (c) PARP1 cleavage (PARPcl) was evaluated at the same time points by western blot analysis, using and anti‐PARP1 antibody. Actin (ACTB) was used as loading control and one representative experiment out of three is shown. The histograms represent the mean plus S.D. of the densitometric analysis of the ratio of cleaved‐PARP1 (PARPcl)/ACTB of three different experiments. (d) DNA content of BC3 and BCBL1 cells treated or not (CT) with Lovastatin (30 μM) for 24 and 48 h was measured with Propidium Iodide staining and analyzed by flow cytometry. The percentage of sub‐G1 events is reported. FACS plots are representative of at least three independent experiments

We first investigated whether Lovastatin, known to display anti‐cancer activity against several cancers, could be cytotoxic against PEL cells. At this aim, BC3 and BCBL1 PEL cell lines were treated with different doses of this drug for 24 and 48 h, based on previous reported studies.^28^, ^29^ As shown in Figure 1a, b, we observed a dose‐ and time‐dependent reduction of cell survival following Lovastatin treatment, in both PEL cell lines. We then found that the impairment of cell survival was due to the induction of apoptosis, as indicated by the cleavage of Poly (ADP‐ribose) polymerase 1 (PARP1) (Figure 1c) and the increase of subG1 events (Figure 1d) in Lovastatin‐treated BC3 and BCBL1 cells. All together these results indicate that Lovastatin reduces the survival of this aggressive lymphoma by triggering an apoptotic cell death.

### 3.2 Lovastatin activates ERK1/2 by reducing STAT3 tyrosine phosphorylation in PEL cells

**FIGURE 2:**
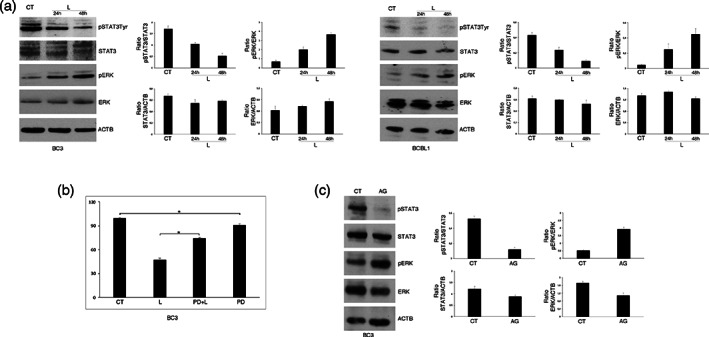
STAT3 inhibition results in ERK1/2 activation after Lovastatin treatment of PEL cell lines. (a) BC3 and BCBL1 cells were treated or not (CT) with Lovastatin (L) (30 μM) for 24 and 48 h and the activation rate of STAT3 (pSTAT3Tyr705) and ERK1/2 (pERK) was evaluated by western blotting. (b) BC3 cells were pre‐treated with PD98059 (PD) (20 μM) and cultured in the presence of Lovastatin (30 μM) for 48 h. Cellular viability was assessed by Trypan blue exclusion. % of cell survival is shown. * < 0.05. (c) PhospoSTAT3Tyr705 and phosphoERK1/2 (pERK) level in BC3 cultured in the presence of AG490 (AG). In figure, Actin (ACTB) was used as loading control and one representative experiment out of three is shown. The histograms represent the mean plus SD of the densitometric analysis of the ratio of ratio of pSTAT3Tyr705/STAT3, STAT3/ACTB, pERK/ERK, and ERK/ACTB of three different experiments

Searching for the molecular pathways affected by Lovastatin treatment, we found that it activated ERK1/2 in both BC3 and BCBL1 cells, in a time‐dependent fashion (Figure 2a). The role of ERK1/2 in PEL cell survival was then assessed by pre‐treating PEL cells with PD98059 ERK1/2 inhibitor before exposure to Lovastatin. As shown in Figure 2b, PD98059 partially rescued cell survival, suggesting that ERK1/2 activation was involved in the cytotoxic effect of Lovastatin. We then found that Lovastatin inhibited STAT3 tyrosine 705 phosphorylation and that this effect contributed to ERK1/2 inhibition, as AG490 STAT3 inhibitor was able to activate ERK1/2 (Figure 2c). The negative regulation of ERK1/2 by STAT3, that was observed here in the course of Lovastatin treatment, has been recently reported in other cancer cell type.^30^

### 3.3 Lovastatin reduces autophagy in PEL cells through ERK 1/2 activation

**FIGURE 3:**
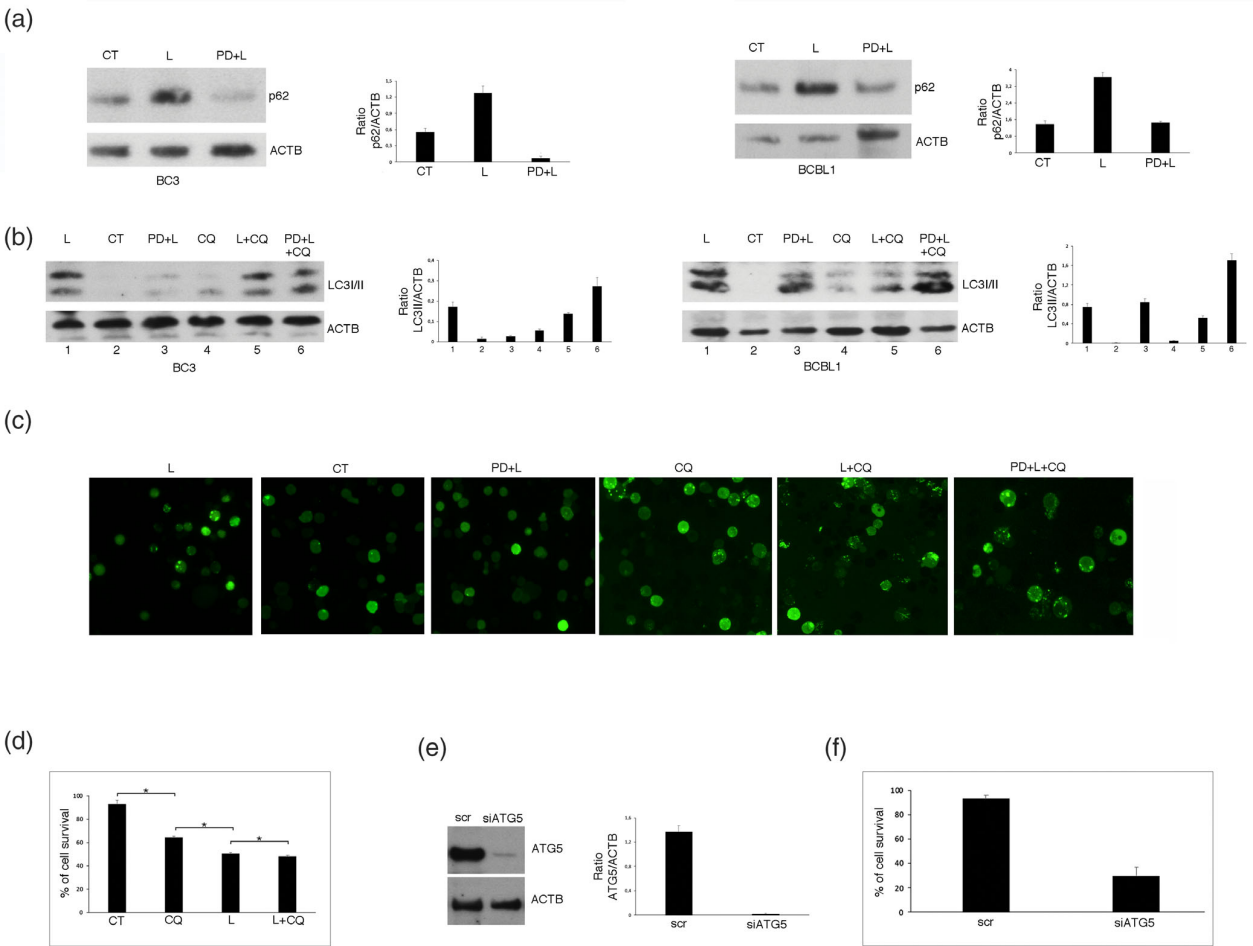
ERK1/2 activation impairs autophagy during Lovastatin treatment of PEL cell lines. Western blotting to assess the expression level of the autophagic markers (a) p62/SQSTM1 (p62) in the presence or absence of PD98059 (PD) (20 μM) and (b) LC3II in the presence or absence of PD98059 (PD) (20 μM) and/or Cloroquine (CQ) (10 μM), in BC3 and BCBL1 cells treated with Lovastatin (L) (30 μM) or vehicle (CT) for 48 h. (c) BC3 cells stably expressing GFP‐LC3 were cultured and treated as in (a) and (b) and LC3‐dots, indicating autophagosomes, were visualized by fluorescence microscopy, at ×20 magnification. (d) BC3 cells were cultured with Lovastatin (L) (30 μM) for 48 h, in the presence or absence of Cloroquine (CQ) and cellular viability assessed by Trypan blue exclusion. % of cell survival is shown. * < 0.05. (e) Western blot analysis to determine ATG5 level in BC3 cells silenced with a specific ATG5 siRNA or a scrambled siRNA (scr). (f) Cellular viability of BC3 cells, after ATG5 silencing, evaluated by Trypan blue exclusion. % of cell survival is shown. * < 0.05. In the figure, Actin (ACTB) was used as loading control and one representative experiment out of three is shown. The histograms represent the mean plus S.D. of the densitometric analysis of the ratio of p62/ACTB, LC3II/ACTB, and ATG5/ACTB of three different experiments

We then evaluated the impact of Lovastatin on autophagy in PEL cells and role of ERK1/2 activation in this process. At this aim, two autophagic markers, p62/SQSTM1 and Microtubule‐associated protein 1A/1B‐light chain 3 (LC3), were analysed by western blot. We found that Lovastatin increased p62/SQSTM1 level in both PEL cell lines (Figure 3a) and induced an accumulation of LC3II that did not increase by combining Lovastatin with Cloroquine (Figure 3b), a drug able to alter lysosomal acidity. These results suggest that Lovastatin inhibits the autophagic flux and that such inhibition occurs at final steps.

To evaluate the role of ERK1/2 on autophagy, we inhibited it by using PD98059 before exposure to Lovastatin and observed that autophagy was restored in both BC3 and BCBL1 cells (Figure 3a, b). This indicates that Lovastatin blocks autophagy through ERK1/2 activation in PEL cells. Besides, we confirmed the results obtained by western blotting using GFP‐LC3‐transfected BC3 cells, in which we observed that Lovastatin induced an accumulation of GFP‐LC3 puncta, indicating autophagosomes, whose number did not increase in the presence of Cloroquine (Figure 3c; L and L + CQ, respectively). Furthermore, the pre‐treatment with PD98059 restored autophagy since GFP‐LC3 puncta increased in these cells in the presence of Cloroquine compared to cells treated with PD98059 and Lovastatin (Figure 3c; PD + L + CQ and PD + L, respectively). We then investigated whether the reduction of autophagy by Lovastatin could contribute to its‐mediated reduction of cell survival. At this aim we blocked autophagy by using Cloroquine or by silencing Autophagy protein 5 (ATG5), an essential autophagic protein^31^ in BC3 cells. As shown in Figure 3d‐f, both treatments reduced PEL cells survival. These results suggest that the inhibition of autophagy contributes to cell death induced by Lovastatin and that this occurs through ERK1/2 activation.

### 3.4 ERK 1/2 engages a cross‐talk with p53 to up‐regulate p21 in PEL cells

**FIGURE 4:**
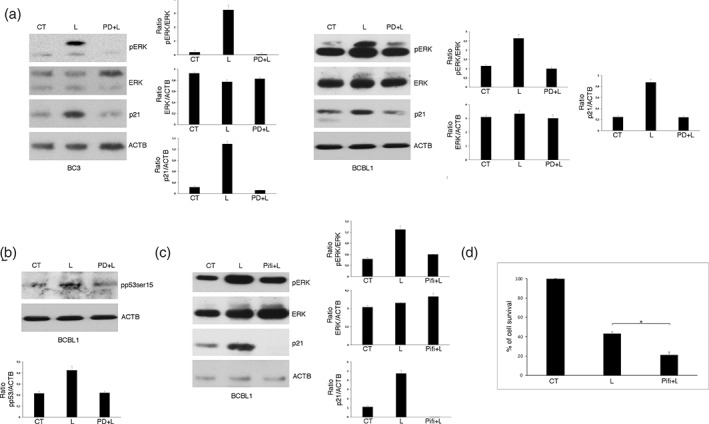
A cross‐talk between ERK1/2 and p53 results in p21 upregulation. (a) BC3 and BCBL1 cell lines were cultured with Lovastatin (L) (30 μM) for 48 h, in the presence or absence of PD98059 (PD) and ERK1/2 activation (pERK) level as well as p21 expression were assessed by western blotting. (b) Ser15 phosphorylation of p53 evaluated by western blotting analysis of BCBL1 cells cultured as in A. (c) ERK1/2 phosphorylation (pERK) and p21 expression level of BCBL1 cells pre‐treated with Pifithrin (Pifi) (20 μM) and then cultured with Lovastatin (L) (30 μM) for 48 h. (d) Cellular viability was evaluated by Trypan blue exclusion. % of cell survival is shown. * < 0.05. In the figure, Actin (ACTB) was used as loading control and one representative experiment out of three is shown. The histograms represent the mean plus SD of the densitometric analysis of the ratio of pERK/ERK and ERK/ACTB and p53Ser15/ACTB of three different experiments

It has been shown that ERK1/2 may up‐regulate p21 expression, depending or not on p53 activation.^22^, ^23^, ^24^ Therefore, we next evaluated p21 expression level in PEL cells treated with Lovastatin in the presence or in the absence of PD98059. As shown in Figure 4a, concomitantly with ERK1/2 activation, p21 was up‐regulated in both BC3 and BCBL1 cells undergoing Lovastatin treatment and that PD98059 counteracted such effect. These results indicate that Lovastatin was up‐regulating p21 also through ERK1/2 activation.

As p21 may be up‐regulated by p53, we then assessed if Lovastatin could activate it, evaluating Ser15 phosphorylation of p53, as this residue may be phosphorylated by ERK1/2. As shown in Figure 4b, p53‐Ser15 phosphorylation, known to play the major role in p21 transcription, increased following Lovastatin treatment and, interestingly, PD98059 counteracted such effect, highlighting the role of ERK1/2 in activating p53‐p21 axis.

To further explore the role of p53 in the up‐regulation of p21, we used Pifithrin‐α, a p53 inhibitor, and found that it counteracted p21 up‐regulation (Figure 4c), highlighting the role of p53 in regulating its expression. Interestingly, Pifithrin‐α also reduced ERK1/2 phosphorylation, which suggests that p53, once activated by ERK1/2, contributed to maintain it activated (Figure 4c). The role of p53 activation in cell survival was then evaluated and, as shown in Figure 4d, we found that Pifithrin‐α further reduced cell survival of Lovastatin‐treated PEL cells. All together these findings indicate that Lovastatin activates ERK1/2 that phosphorylates Ser15 of p53 that in turn up‐regulates p21 expression level to sustain cell survival and maintain ERK1/2 activated in PEL cells.

### 3.5 p21 inhibitor UC2288 increases PEL cell death induced by lovastatin

**FIGURE 5:**
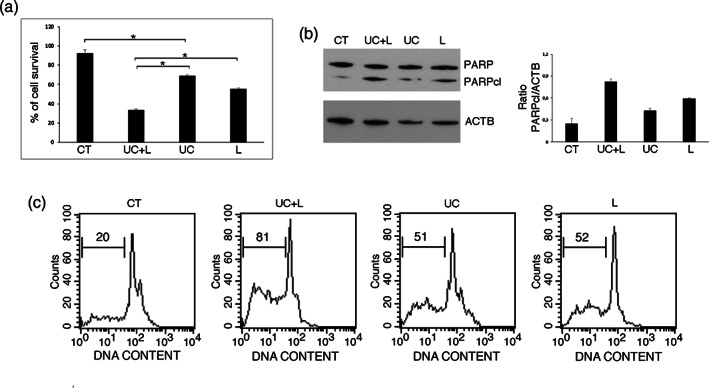
Lovastatin‐induced cell death increases after p21 inhibition with UC2288. BCBL1 cells were pre‐treated with UC2288 (UC) and then cultured with Lovastatin (L) (30 μM) for 48 h and (a) cellular viability was evaluated by Trypan blue exclusion. % of cell survival is shown. * < 0.05; (b) PARP1 cleavage (PARPcl) was assessed by western blotting; (c) DNA content was measured with Propidium Iodide staining and analyzed by flow cytometry. The percentage of sub‐G1 events is reported. FACS plots are representative of at least three independent experiments. The Student *t* test was used for statistical significance of the differences between treatment groups. Statistical analysis was performed using analysis of variance at 5% (*p* < .05). The Student *t* test was used for statistical significance of the differences between treatment groups. Statistical analysis was performed using analysis of variance at 5% (*p* < .0)

p21 may have a double effect in cancer,^32^ as it blocks cell cycle and arrest growth following DNA damage in one hand while, on the other hand, it may act as an inhibitor of p53‐dependent apoptosis. Therefore, we then inhibited p21 by using UC2288 in combination with Lovastatin and evaluated the role of its up‐regulation in Lovastatin‐induced cytotoxicity against PEL cells. As shown in Figure 5a, UC2288, that per se induced a cytotoxic effect against PEL cells, increased cell death induced by Lovastatin. This suggests that p21 up‐regulation induced by the activation of ERK1/2‐p53 axis was promoting cell survival in this setting. According to the reduction of cell survival, we found that UC2288 increased Lovastatin‐induced PARP cleavage (Figure 5b) and the number of subG1 events (Figure 5c) in PEL cells, confirming that UC2288 was potentiating the apoptotic effect of Lovastatin against PEL. These data, evidencing that ERK1/2 activation by Lovastatin was also somehow sustaining cell survival, could explain why PD98059 pre‐treatment partially but not totally reverted the cytotoxic effect of Lovastatin on PEL cells.

## 4 DISCUSSION

The search for new and more effective therapies against PEL is needed, given its poor response to anti‐cancer therapies and its aggressive behavior.^33^ Here, we found that Lovastatin efficiently induced apoptosis in PEL cells, in dose‐ and time‐dependent fashion. Lovastatin as other Statins has been reported to mediate an anti‐cancer effect.^1^ This is because it targets lipid metabolism or the mevalonate pathway that is dysregulated in many cancer types,^34^, ^35^ because inhibits several pro‐survival pathways and may, in some case, act as a proteasome or HDAC inhibitor.^1^ At molecular level, in this study, we showed that Lovastatin activated ERK1/2 in PEL cells, effect quite unexpected, as the majority of previous studies have reported that Statins inhibited ERK1/2 rather than activating it.^36^, ^37^ The Lovastatin‐mediated ERK1/2 activation correlated with de‐phosphorylation of STAT3 Tyr705, pathway constitutively activated in PEL and essential for its survival.^16^, ^17^, ^18^ Indeed, the AG490 JAK2/STAT3 inhibitor activated ERK1/2. Several studies have reported that ERK1/2 activation could reduce STAT3 Tyr705 phosphorylation^38^, ^39^ while other studies demonstrated that STAT3 inhibition could increase ERK1/2 activation,^30^, ^40^ as we observed here. However, differently from these studies, the activation of ERK1/2 by STAT3 inhibition contributed to the cytotoxic effects of Lovastatin in PEL cells rather than preventing them. This may be also because the activation of ERK1/2 resulted in a block of autophagy, process important for PEL cell survival as demonstrated by the use of Cloroquine or by the silencing of TG5. Interestingly, ERK1/2 has been reported to have opposite roles in cancer: its activation has indeed been shown to either promote or inhibit cancer cell proliferation, depending on the intensity and duration of ERK signaling and on its interplay with other signaling pathway.^41^ The intensity and duration of ERK1/2 activation seems also to influence autophagy, as for example its prolonged activation has been reported to reduce the autophagic flux^26^ while, in pancreatic cancer, ERK1/2 inhibition de‐regulated metabolic activity and promoted autophagy.^42^ Autophagy inhibition by ERK1/2 has been reported to occur at the last autophagic steps in Bortezomib‐treated ovarian cancer or in Marsdenia tenacissim‐treated lung cancer cells. Indeed, the activation of ERK1/2 resulted in the reduction of cathepsin B in both cases and LAMP1 downregulation in the latter case.^43^, ^44^ In future studies, it will be interesting to investigate whether Lovastatin could influence cathepsin B and/or LAMP1 expression level. Of note, autophagy is strictly inter‐connected with apoptosis^45^ and it is possible that different impact of ERK1/2 activation on prevention or induction of cell proliferation may depend on its different regulation of autophagy. Among the molecules with which ERK1/2 interacts, there are both mutp53^46^ and wtp53. In the latter case, ERK1/2 induces the activation of wtp53 that, in turn, promotes the transcription of targets that oppositely regulate growth arrest‐survival and cell death.^47^ In particular, the transcription of p21, a p53 target gene, may sustain survival or promote cell death, depending on its subcellular localization.^32^ Indeed, when p21 localizes in the cytoplasm, it acts as an oncogene, while in its nuclear localization it acts as tumor suppressor.^48^ In the first case, when p21 promotes cell proliferation, its targeting by small inhibitors seems to be an efficient strategy to reduce tumor growth.^21^ ERK1/2 may increase the expression of p21 through ser15 p53 phosphorylation as well as through other mechanisms.^23^, ^24^ Here we found that p21, up‐regulation by p53 activated by ERK1/2, sustained PEL cell survival in the course of Lovastatin treatment. Indeed, targeting p21 by UC2288, that per se reduced PEL cell survival, increased the cytotoxic effect of Lovastatin against these cells.

In conclusion, this study suggests that Lovastatin could represent a new therapeutic strategy against PEL and that the cytotoxic effect of this drug could be improved by p21 using inhibitors. The latter finding is important as in vivo studies have suggested that the anti‐cancer effects of Lovastatin is observed when used in combination with other treatments.^49^

## CONFLICT OF INTEREST

The authors declare no conflicts of interest.

## Data Availability

The data that support the findings of this study are available from the corresponding author upon reasonable request.
